# The management of patients who self-harm in adult inpatient mental health settings: A policy analysis of English NHS mental health trusts

**DOI:** 10.1371/journal.pone.0327358

**Published:** 2025-07-01

**Authors:** Hannah Sharp, Judith Johnson, Cathy Brennan, John Baker

**Affiliations:** 1 School of Healthcare, University of Leeds, Leeds, United Kingdom; 2 School of Health Sciences, University of Manchester, Manchester, United Kingdom; 3 Leeds Institute of Health Sciences, University of Leeds, Leeds, United Kingdom; Jouf University, SAUDI ARABIA

## Abstract

The management of self-harm is a critical focus for staff in inpatient mental health settings. This study aimed to better understand how staff are guided through policies to manage self-harm via the following objectives: 1) to assess the alignment of policies from English NHS Mental Health trusts with national guidelines, 2) identify which aspects of the national guidelines are most and least frequently reflected in these policies, and 3) determine whether trusts with dedicated self-harm policies better reflect national guidelines. We conducted a content analysis of self-harm-related policies across 50 English NHS mental health trusts against a framework of 20 standards created from National Institute for Health and Care Excellence self-harm guidelines. Our analysis revealed a significant difference (U = 36.50, *p* = .002) in the number of standards met by trusts with a specific self-harm policy (*M* = 11.44, *SD* = 3.00) compared to those without (*M* = 7.26, *SD* = 3.00), with the number of standards met ranging from zero to 15. Notably, trusts failed to meet the majority of standards (M = 11.69, SD = 3.30). The findings of this study highlight several new insights into NHS trust policy on self-harm: 1) trusts exhibit variability in how they organise information across their policies, 2) dedicated self-harm policies may support trusts to better meet guidance but risk complicating guidance for staff, 3) policy content varies across trusts, 4) the importance of patient voice is acknowledged but the facilitators of good participation are poorly supported in the same policies, 5) trusts rarely define self-harm and some trusts use definitions which do not reflect guidelines, and 6) harm-reduction remains underrepresented in policies, reflecting ongoing contention surrounding its implementation. Further research is needed to understand the role that policy and guidelines play in guiding staff practices when managing self-harm.

## Introduction

Self-harm is a significant issue for mental health services. Between 2015 and 2022, English mental health services, across both community and inpatient settings, experienced a 95% increase in reported incidents of self-harm [1]. Furthermore, the number and rate of mental health inpatient suicides in the UK (excluding Northern Ireland) has not fallen since 2016 [2].Of the 1,052 inpatient deaths by suicide in the UK between 2011 and 2021, 28% of those patients died whilst under ‘enhanced nursing observations’ meaning they were deemed by their service to be at a higher risk of self-harm and/or suicide [2]. This data is indicative of a system-wide need to consider self-harm as growing patient safety issue.

The approach taken to managing self-harm is of particular importance within inpatient mental health environments, where the approach taken can present risks to both patients and staff. For example, restraint, seclusion, and coerced intramuscular medication have all been identified as practices which are used in these environments to manage self-harm [3–5] and are known to carry the potential to cause both physical and psychological harm to patients and staff [6–8]. The data highlighted above relating to inpatient suicides under enhanced observations is also indicative that other approaches currently taken to manage self-harm are not consistently effective.

Existing research suggests that focusing on the inpatient environment itself, including both a focus on its therapeutic nature and the safety of the physical environment, may have the potential to improve patient safety [2]. There is also evidence that how healthcare staff choose to approach managing self-harm is influenced by their attitudes towards it [9]. One mechanism that has the potential to impact on all of these areas is the policies mental health services expect staff to follow. Not only does policy provide services with the opportunity to outline how they should operate regarding issues such as maintaining environmental safety, but staff also report that their attitudes towards self-harm can be impacted via the practice guidelines they are given which influence how equipped they feel to manage it effectively [10].

In large health systems, such as the English National Health Service (NHS), responsibility for certain elements of decision-making regarding service delivery is often delegated to a number of regional and local organisations. These organisations may further delegate decisions regarding operational and clinical management to individual providers. In the case of the NHS, these providers are often NHS trusts [11].

Whilst NHS trusts have a degree of freedom to develop their own policies and procedures, national guidelines, such as those produced by the National Institute for Health and Care Excellence (NICE), do set out standards of good practice. These trusts ‘must have due regard to’ these guidelines, though they do carry a ‘non-mandatory’ status [12].

The freedom NHS trusts have when setting their own policies leaves scope for services to adapt to better meet the needs of their local population. It also, therefore, leaves scope for a variety of approaches to the management of self-harm. A scoping review covering suicide risk assessment in a variety of both mental health-focused and non-mental health-focused NHS settings indicated that such variation does exist in practice [13]. There is also evidence that the use of some of the approaches which deviate from NICE guidelines may have negative impacts on patient experience and safety [14,15]. A lack of similar research on how trusts manage self-harm means insight into how staff are being guided to manage it remains limited. This context means improving our understanding of the approaches trusts direct staff to take to manage self-harm is a crucial step in the identification of opportunities for systemic improvements relating to patient experience and safety.

Taking the NICE 2004 and 2011 guidelines on the short- and long-term management of self-harm [16,17], this study has developed a set of standards against which to assess whether the policies produced by English NHS trusts commissioned to provide secondary mental health care (hereafter referred to as ‘mental health trusts’) reflect what is broadly considered to be best-practice for managing self-harm. Our objectives were to 1) establish the extent to which the policies produced by English NHS Mental Health trusts align with the NICE guidelines, 2) identify the areas of the NICE guidelines which are more or less frequently reflected in those policies, and 3) investigate whether trusts with a specific self-harm policy better reflect the NICE guidelines than those without such a policy.

## Methods

### Design

This study conducted a content analysis on policies obtained from English NHS Mental Health trusts via freedom of information requests. The research process used a list of factors derived from the 2011 and 2004 NICE guidelines on the long-term and short-term management of self-harm to guide data extraction and analysis. These guidelines were chosen as they were produced via ‘systematic reviews of best available evidence’, expert consensus, and patient and public involvement [18]. They contain a range of recommendations relating to the management of self-harm across all healthcare settings, including attending to both the physical and psychological health needs of patients presenting with self-harm. Whilst new guidelines were published prior to this analysis [19] it was deemed that, as the data collection began 3 months after their publication, services could not be expected to have updated their policies within that timeframe to reflect the new guidelines.

### Self-harm definition

Various terms for ‘self-harm’ are often used interchangeably but can be distinctly defined. Self-mutilation [20], non-suicidal self-injury [21], parasuicide [22], and self-poisoning [23] are just some of the other terms used which come under the umbrella of acts where an individual causes harm to themselves.

‘Self-harm’ in this study was defined by the NICE 2011 guideline as:

“any act of self-poisoning or self-injury carried out by an individual irrespective of motivation.”

This study’s definition is therefore also inclusive of attempted suicide and suicide.

### Inclusion and exclusion criteria

Policies relating to the management of self-harm, as defined above, in the adult inpatient environment were included in this study. The selected policies comprised of policies which focused explicitly on self-harm. This included policies which were presented as focusing on ligatures, suicide, and clinical risk management. Where policies did not make clear whether they referred specifically to inpatients, it was taken that they were inclusive of all patients in the trust and these policies were therefore included. Policies which referred only to children and adolescents, only to older adults, or only to outpatients were excluded. Whilst suicide was included in the definition of self-harm used in this study, the NICE 2004 and 2011 guidelines focus on management of self-harm in reference to supporting the individual who has self-harmed or is at risk of self-harm. Therefore, policies which only referred to the management of an inpatient suicide (e.g. a learning from deaths policy) were also excluded. The suicide policies included in this study therefore focused on suicide prevention.

### Procedure

Freedom of Information requests were sent on 14/12/2022 to 50 mental health trusts in England.. The number of trusts providing adult inpatient mental health services does fluctuate and the 50 trusts contacted included all trusts identifiable via internet searches that provided adult inpatient mental health services at the time of the request. The request asked for “copies of all policies which refer to the management of self-harm or suicidal patients in inpatient settings”. Freedom of Information requests were used as not all trusts publish these policies publicly. Thirty-five working days were given for trusts to respond to the request.

### Data extraction and analysis

The research team synthesised the standards in the NICE guidelines via an iterative process of drafting and discussion. Twenty standards were agreed upon which covered the parts of the guidelines which were applicable to inpatient mental health environments, and content analysis of the policies was conducted using these as a framework. These standards are listed in Table 1.

**Table 1:** Standards extracted from the NICE 2004 and 2011 guidelines.

Standard
***The policy:***
(1) Aligns with the 2011 NICE definition of self-harm
(2) Highlights the need for staff to develop a ‘trusting and supportive’ relationship with service users
(3) Advocates for shared decision-making
(4) Outlines that full written and verbal information on the treatment options for self-harm should be provided to service-users
(5) Outlines the provision of appropriate training – including specifically on the management of self-harm
(6) Stipulates that training must be co-produced and use outcome measures with patients to assess the training’s effectiveness
(7) Stipulates that routine supervision must take place
(8) Outlines that the service user ought to be consulted about family involvement
(9) Outlines that transitions or transfers of care ought to be planned for in advance
(10) Stipulates that a full assessment of needs must take place
(11) Outlines that the meaning of the self-harm is discussed with the patient during assessment
(12) Outlines that a full risk assessment must take place in line with the content of the 2011 NICE guidelines
(13) Highlights that risk assessment tools should not be used to predict future suicide or SH, but they can be used to help structure assessment
(14) Stipulates that the care plan and risk management plan are developed in conjunction with the patient (and family and/or significant others if this is consented to by the patient)
(15) Stipulates that the risk management plan must be regularly updated
(16) Stipulates that the care plan must be reviewed at intervals of no more than one year
(17) Does not recommend the use of drug treatment specifically for SH
(18) Outlines that each episode of self-harm should be treated in its own right
(19) Outlines that clinicians should provide appropriate advice on harm-reduction
(20) Outlines that service users should always be offered physical treatment which is carried out in line with the 2011 NICE guidelines

In the first round of analysis, the policies specifying a focus only on ‘self-harm’ were reviewed and data were extracted into a spreadsheet where trusts were marked as having a policy which either met a standard (yes), contradicted it (no), or did not contain any relevant information (DNC). Policies labelled as suicide-related, ligature policies, and clinical risk management policies were then also subjected to the same analysis. Throughout the analysis, the original standards from the NICE guidelines were referred to in order to guide whether a policy was said to fulfil the criteria of the relevant standard. Regular discussion also took place amongst three members of the research team (HS, JJ, and JB) throughout this process to review the interpretation of the contents of policy documents and ensure that the standards were applied consistently across the documents included. This data was then compiled to identify the number of trusts meeting each standard, as well as which trusts met which standards. This allowed us to identify which standards were more frequently and less frequently met overall, across all relevant policies provided by trusts.

Once marked against the standards, a Shapiro-Wilk test was performed to check the distribution of the number of standards met by trusts with and without a specific self-harm policy. The test showed that this distribution departed significantly from normality for trusts without a specific self-harm policy (W(26) = 0.88, p = 0.01). A non-parametric test, the Mann-Whitney U test, was therefore chosen as most appropriate for the analysis of the data in line with the third aim of this study.

## Results

### Descriptive information

In the 35 days allotted 45 (90%) of trusts responded. The five trusts which did not respond did not give a justification for not responding to the request. Ten of the 45 trusts that responded (22%) did not provide any policy meeting inclusion criteria. Thirty-five (78%) of the responding trusts therefore provided at least one policy meeting the inclusion criteria. The ten trusts responding which did not provide at least one policy meeting inclusion criteria either responded only with policies not meeting the inclusion criteria (n = 7) or indicated that they had policies which met the criteria but could not disclose them due to patient safety concerns (n = 3). Patient safety concerns were also cited by two other trusts which withheld policies eligible for inclusion, however these trusts provided other policies which did meet the criteria for the study.

The policies identified for inclusion in the study comprised of nine policies which specifically focused on self-harm, six suicide-related policies, nine ligature management policies, and 31 clinical risk management policies. The study also included one ‘clinical care policy’ which also covered other areas of care however had two defined sections which focused on ligature and clinical risk management. These two sections of the policy were deemed to meet inclusion criteria and so only these sections were used for data extraction.

Of the 56 relevant policies provided, ten (18%) policies were out-of-date across ten trusts and a further four (7%) policies from four trusts did not specify a review date. Two trusts specified that their out-of-date policies were under review. The most out-of-date policy was 18-months past its review date at the time of request. No reasons were provided for delays in reviewing any of the out-of-date policies.

Policies also varied in length, with the self-harm-specific policies ranging from six to 32 pages in length (*M *= 20.25, *SD* = 8.29). The longest policy was the clinical care policy which comprised of 643 pages however the sections meeting inclusion criteria had a combined length of 20 pages.

### Policy content information

The content of policies also varied. The number of standards met by each trust ranged from zero to 15 (*M *= 8.34, *SD *= 3.30) out of a possible 20 standards. There was a significant difference (U = 36.50, *p* = .002) between the number of standards met by the nine trusts with a specific self-harm policy and the 26 without a specific self-harm policy that provided other policies which met the inclusion criteria. The mean number of standards met by trusts with a specific self-harm policy was 11.44 (*SD *= 3.00) compared with 7.27 (*SD* = 2.69) by those without, suggesting that trusts with a specific self-harm policy are more likely to reflect more of the NICE guidelines.

Table 2 outlines the number of trusts which met, contradicted, or did not contain the relevant information to meet each standard. Table 3 contains the anonymised results for each trust, demonstrating the variability in how many standards each trust met, contradicted, or failed to contain the relevant information to meet.

**Table 2:** How many trusts met or contradicted each standard, or the relevant information was absent – overview.

Standard	Number of Trusts where:		
*The policy:*	*One of the assessed policies met this standard (Yes)*	*No policy contained information relevant to this standard (DNC)*	*Information in a policy directly contradicted the standard (No)*
(1) Aligns with the 2011 NICE definition of self-harm	4	29	2
(2) Highlights the need for staff to develop a ‘trusting and supportive’ relationship with service users	14	21	0
(3) Advocates for shared decision-making	31	4	0
(4) Outlines that full written and verbal information on the treatment options for self-harm should be provided to service-users	6	29	0
(5) Outlines the provision of appropriate training – including specifically on the management of self-harm	4	31	0
(6) Stipulates that training must be co- produced and use outcome measures with patients to assess the training’s effectiveness	2	33	0
(7) Stipulates that routine supervision must take place	30	5	0
(8) Outlines that the service user ought to be consulted about family involvement	17	18	0
(9) Outlines that transitions or transfers of care ought to be planned for in advance	15	20	0
(10) Stipulates that a full assessment of needs must take place	29	6	0
(11) Outlines that the meaning of the self-harm is discussed with the patient during assessment	2	33	0
(12) Outlines that a full risk assessment must take place in line with the content of the 2011 NICE guidelines	30	5	0
(13) Highlights that risk assessment tools should not be used to predict future suicide or self-harm, to determine who should and should not be offered treatment, or to determine who should be discharged. They can be used to help structure assessment	24	7	4
(14) Stipulates that the care plan and risk management plan are developed in conjunction with the patient (and family and/or significant others if this is consented to by the patient)	30	5	0
(15) Stipulates that the risk management plan must be regularly updated	32	3	0
(16) Stipulates that the care plan must be reviewed at intervals of no more than one year	7	28	0
(17) Does not recommend the use of drug treatment specifically for SH	1	34	0
(18) Outlines that each episode of self-harm should be treated in its own right	7	28	0
(19) Outlines that clinicians should provide appropriate advice on harm-reduction	3	32	0
(20) Outlines that service users should always be offered physical treatment which is carried out in line with the 2011 NICE guidelines	4	31	0

**Table 3:** How many trusts met or contradicted each standard, or the relevant information was absent – by trust.

Trust	Standard																				Number of standards marked as		
	1	2	3	4	5	6	7	8	9	10	11	12	13	14	15	16	17	18	19	20	Yes	DNC	No
1	DNC	DNC	Yes	DNC	DNC	DNC	Yes	Yes	DNC	Yes	DNC	Yes	Yes	Yes	Yes	DNC	DNC	Yes	DNC	DNC	9	11	0
2	DNC	DNC	Yes	DNC	DNC	DNC	Yes	DNC	Yes	Yes	DNC	Yes	Yes	Yes	Yes	DNC	DNC	DNC	DNC	DNC	8	12	0
3	DNC	Yes	Yes	DNC	DNC	DNC	Yes	DNC	DNC	Yes	DNC	Yes	No	DNC	Yes	DNC	DNC	DNC	DNC	DNC	6	13	1
4*	Yes	Yes	Yes	DNC	DNC	DNC	Yes	DNC	DNC	Yes	DNC	DNC	Yes	Yes	Yes	Yes	DNC	Yes	Yes	DNC	11	9	0
5	DNC	DNC	Yes	DNC	DNC	DNC	Yes	Yes	Yes	Yes	DNC	Yes	Yes	Yes	Yes	DNC	DNC	DNC	DNC	DNC	9	11	0
6	DNC	DNC	DNC	DNC	DNC	DNC	DNC	DNC	DNC	DNC	DNC	DNC	DNC	Yes	Yes	Yes	DNC	DNC	DNC	DNC	3	17	0
7*	Yes	Yes	Yes	DNC	DNC	DNC	Yes	Yes	Yes	Yes	DNC	Yes	Yes	Yes	Yes	DNC	Yes	Yes	Yes	Yes	15	5	0
8	DNC	Yes	Yes	DNC	DNC	DNC	Yes	DNC	Yes	Yes	DNC	Yes	DNC	Yes	Yes	Yes	DNC	Yes	DNC	DNC	10	10	0
9*	No	Yes	Yes	DNC	DNC	DNC	Yes	Yes	Yes	Yes	DNC	Yes	No	Yes	Yes	DNC	DNC	DNC	DNC	DNC	9	9	2
10	DNC	DNC	Yes	DNC	DNC	DNC	Yes	DNC	DNC	Yes	DNC	Yes	Yes	Yes	Yes	DNC	DNC	DNC	DNC	DNC	7	13	0
11	DNC	DNC	Yes	DNC	DNC	DNC	DNC	Yes	Yes	Yes	DNC	Yes	No	Yes	Yes	DNC	DNC	DNC	DNC	DNC	7	12	1
12	DNC	DNC	Yes	DNC	DNC	DNC	Yes	Yes	Yes	Yes	DNC	Yes	Yes	Yes	Yes	DNC	DNC	DNC	DNC	DNC	9	11	0
13	DNC	DNC	Yes	DNC	DNC	DNC	Yes	DNC	DNC	Yes	DNC	Yes	No	Yes	Yes	DNC	DNC	DNC	DNC	DNC	6	13	1
14	DNC	DNC	Yes	Yes	DNC	DNC	Yes	DNC	DNC	Yes	DNC	Yes	Yes	Yes	Yes	Yes	DNC	DNC	DNC	DNC	9	11	0
15	DNC	DNC	DNC	DNC	DNC	DNC	DNC	DNC	DNC	DNC	DNC	DNC	DNC	DNC	Yes	DNC	DNC	DNC	DNC	DNC	1	19	0
16	DNC	DNC	Yes	DNC	DNC	DNC	Yes	DNC	DNC	Yes	DNC	DNC	Yes	Yes	Yes	DNC	DNC	DNC	DNC	DNC	6	14	0
17	DNC	DNC	Yes	Yes	DNC	DNC	Yes	Yes	DNC	Yes	DNC	Yes	Yes	Yes	Yes	DNC	DNC	DNC	DNC	DNC	9	11	0
18	DNC	Yes	Yes	DNC	DNC	DNC	Yes	DNC	DNC	Yes	DNC	Yes	Yes	Yes	Yes	DNC	DNC	DNC	DNC	DNC	8	12	0
19*	Yes	Yes	Yes	Yes	DNC	Yes	DNC	Yes	Yes	Yes	Yes	Yes	Yes	Yes	DNC	DNC	DNC	Yes	DNC	Yes	14	6	0
20*	No	Yes	Yes	Yes	DNC	Yes	Yes	DNC	DNC	Yes	DNC	Yes	Yes	Yes	Yes	DNC	DNC	Yes	Yes	Yes	13	6	1
21*	DNC	DNC	Yes	DNC	DNC	DNC	Yes	Yes	DNC	Yes	DNC	Yes	DNC	Yes	Yes	DNC	DNC	DNC	DNC	DNC	7	13	0
22	DNC	DNC	DNC	DNC	DNC	DNC	Yes	DNC	Yes	Yes	DNC	Yes	Yes	DNC	Yes	DNC	DNC	DNC	DNC	DNC	6	14	0
23	DNC	Yes	Yes	DNC	Yes	DNC	Yes	Yes	DNC	Yes	DNC	Yes	Yes	Yes	Yes	DNC	DNC	DNC	DNC	DNC	10	10	0
24*	DNC	DNC	Yes	Yes	DNC	DNC	Yes	Yes	Yes	Yes	DNC	Yes	Yes	Yes	Yes	Yes	DNC	DNC	DNC	DNC	11	9	0
25	DNC	Yes	Yes	DNC	Yes	DNC	Yes	DNC	Yes	Yes	DNC	Yes	DNC	Yes	Yes	DNC	DNC	DNC	DNC	DNC	9	11	0
26*	DNC	Yes	Yes	DNC	DNC	DNC	Yes	Yes	DNC	Yes	DNC	Yes	Yes	DNC	Yes	DNC	DNC	DNC	DNC	DNC	8	12	0
27	DNC	DNC	Yes	DNC	DNC	DNC	Yes	Yes	Yes	Yes	DNC	Yes	Yes	Yes	Yes	DNC	DNC	DNC	DNC	DNC	9	11	0
28	DNC	DNC	Yes	DNC	DNC	DNC	Yes	Yes	DNC	DNC	DNC	Yes	Yes	Yes	Yes	DNC	DNC	DNC	DNC	DNC	7	13	0
29	DNC	Yes	Yes	DNC	DNC	DNC	Yes	Yes	Yes	DNC	DNC	Yes	DNC	Yes	Yes	Yes	DNC	DNC	DNC	DNC	9	11	0
30	DNC	DNC	Yes	DNC	Yes	DNC	Yes	DNC	DNC	DNC	DNC	Yes	Yes	Yes	DNC	DNC	DNC	DNC	DNC	DNC	6	14	0
31	DNC	DNC	DNC	DNC	DNC	DNC	DNC	DNC	DNC	DNC	DNC	DNC	DNC	DNC	DNC	DNC	DNC	DNC	DNC	DNC	0	20	0
32	DNC	Yes	Yes	DNC	Yes	DNC	Yes	Yes	Yes	Yes	DNC	Yes	Yes	Yes	Yes	Yes	DNC	DNC	DNC	DNC	12	8	0
33	DNC	DNC	Yes	DNC	DNC	DNC	Yes	DNC	DNC	Yes	DNC	Yes	Yes	Yes	Yes	DNC	DNC	DNC	DNC	DNC	7	13	0
34*	Yes	Yes	Yes	Yes	DNC	DNC	Yes	Yes	Yes	Yes	Yes	Yes	Yes	Yes	Yes	DNC	DNC	Yes	DNC	Yes	15	5	0
35	DNC	DNC	Yes	DNC	DNC	DNC	Yes	DNC	DNC	Yes	DNC	Yes	Yes	Yes	Yes	DNC	DNC	DNC	DNC	DNC	7	13	0

Yes = At least one policy met the standard, DNC = No policy contained information relating to the standard, No = Information in at least one policy directly contradicted the standard.

* Trusts with an explicit self-harm policy.

These results highlight that self-harm is rarely defined by English NHS trusts (standard one) and of the six trusts where self-harm was defined, two of these definitions deviated from that within the NICE 2011 guidelines. One of these two trusts chose to explicitly exclude attempted suicide from their definition as they communicated the importance of recognising that not everyone who self-harms does so because they experience suicidal ideation, and that those who experience suicidal ideation are not always self-harming with the intention of a fatal outcome. The second trust did not explicitly exclude attempted suicide from their definition, but instead chose to specify that self-harm is a ‘non-fatal’ act. The NICE 2011 guidelines do not take the fatality or intention of an act of self-harm into consideration when defining it.

The only other standard which saw direct contradiction was standard 13, that risk assessment tools should not be used to predict future suicide or self-harm to determine who should and should not be offered treatment or to determine who should be discharged. Whilst they can be used to help structure assessment under NICE guidelines, four trusts highlighted that risk assessment tools should be used to inform clinical decision-making and care planning. This means that these trusts stipulate that risk assessment tools should be used as part of decisions regarding treatment and discharge. None of these four trusts gave further guidance which highlighted the components of risk assessment beyond the use of their chosen tools and therefore they are the only guidance given to staff via policy on the matter. These trusts were therefore marked as containing information in a policy directly contradicting standard 13.

The most frequently reflected element of the NICE guidelines related to ensuring that patient risk management plans were regularly updated (standard 15) and 30 of the 35 trusts reviewed stipulated that the risk management plan should be developed in conjunction with the patient (standard 14).

## Discussion

This study highlights new findings in relation to the content of the policy portfolios of English NHS Mental Health trusts. It found that, on average, English NHS mental health trust policies contain information which covers less than half (42%) of the NICE 2004 and 2011 guidelines on the management of self-harm. It has also demonstrated the variability in which areas of the NICE guidelines trusts reflect in their policies, with the number of trusts meeting each standard ranging from one (3%) to 32 (91%). It found that trusts which had a specific self-harm policy within their portfolio significantly reflected these guidelines more accurately, but that only nine out of the 45 responding trusts provided such a policy.

It may, therefore, be a fair recommendation that trusts develop specific self-harm policies to ensure their guidance for staff clearly reflects national guidance. This study also demonstrates, however, that self-harm is addressed across multiple policies within a trust’s portfolio which may not specifically label themselves as focusing on ‘self-harm’. When a policy portfolio becomes segmented in this way staff face challenges when they wish to consult policy to guide their practice. For example, a staff member wishing to support a patient who is known to regularly ligature may consult their trust’s ligature policy. They may also find relevant information in the clinical risk management policy. Depending on the intent expressed by the patient, it may also fall under the jurisdiction of a suicide-related policy. If a trust has an additional self-harm policy, this may also contain important information. The variation in the scopes of the policies provided in response to the Freedom of Information requests demonstrates these difficulties further. For some staff, a clinical risk management policy clearly related to the management of self-harm or suicidal patients and therefore it was included in their response to the request. For others, it is possible that their trust had a policy like this but they did not see this as a potential source of information on the topic and so did not provide a copy.

The way guidelines are presented to staff across multiple, overlapping documents has the potential to cause confusion amongst staff and inconsistency in care. The existence of such confusion and inconsistency when it comes to managing self-harm is reflected in other research. For example, a survey of inpatient mental health service staff found that 17.5% of respondents felt that there was a lack of consistency in how self-harm using ligatures was managed within their service [24]. Inconsistent care is known to reduce a patient’s trust in staff, presenting challenges for providing safe and effective care [25]. So, whilst introducing a specific self-harm policy to a trust’s policy portfolio would allow trusts to provide more information to staff, this approach must be carefully considered alongside the risk of increasing the complexity of the already complex information sources staff utilise.

A further finding of this study for discussion was the variability in which standards were more or less often met. Despite this variability, there were areas of the guidelines which trusts met more consistently. The standards on risk management planning (standards 14 and 15) were amongst those met by over 85% of trusts. The decision to admit or discharge a patient to and from the inpatient environment is made based on a number of factors and ‘risk’ is considered to be the factor which dominates this decision-making [26]. Therefore, a primary focus of an admission is often managing a patient’s risk with the aim of reducing it such that they can be discharged. A study by Deering et al., [27] found that doing this effectively requires working collaboratively with the patient over time to build trust and support them to gradually take more responsibility for managing their own risks. On this basis it is understandable that these standards, which focused on regularly updating risk management plans in conjunction with the patient, were amongst the most commonly met.

A recognition of the importance of the voice of the patient in their care was further reflected across the policies included in this study via standard three, that the policy ‘Advocates for shared decision-making’. This was met by 31 of the 35 trusts (89%). Shared decision-making is a core part of person-centred care [28]. Person-centred care is key to treating patients with dignity, compassion, and respect as well as ensuring care is effective, safe, and promotes independence [29]. A recognition of its importance helps the content of policies align with these values and goals. This study does however suggest that greater consideration may still need to be given to ensuring that the factors which facilitate patient involvement are also appropriately supported. Of the trusts meeting standard three, only six also met standard four that the policy ‘outlines that full written and verbal information on the treatment options for self-harm should be provided to service-users’. For shared decision-making to occur, the patient must be provided with the necessary information to participate in discussions about their different care options [30].

Additionally, only two of the trusts meeting standard three also met standard 11 which requires that trusts advocate that the meaning of the self-harm for the patient is explored during their assessment. Including the voice of the patient in this way helps to support person-centred care, the importance of which is highlighted above. Taking this individualised approach would indicate a more collaborative approach, working with the patient to create their formulation. The dialogue such an approach would require indicates an assessment process that would support the service users voice in decision-making.

A further area which trusts may still wish to give consideration to is the inclusion of a consistent definition for self-harm in their policies. 83% of trusts did not provide any definition for self-harm, and 6% of trusts did not use the NICE 2011 definition for it (standard one). Variability in how self-harm is defined across the literature has been identified before [31] but this is the first study to explore how any mental health system defines self-harm within its policies. Without a consistent definition of self-harm being used throughout the health system what a staff member may choose to input into a patients record as an incident of self-harm is left at a greater risk of being influenced by their own interpretation or the interpretations of others. This makes it difficult to be confident that incidents are being reported accurately, clinical records maintained appropriately, and effective care delivered. Care planning and delivery relies on accurate information and a shared-understanding about a patients presentation [32]. There is also evidence that staff understanding of self-harm impacts on their attitudes towards, and their approach to supporting, patients who self-harm [33]. Furthermore, accurate data is important for services when reviewing safety and quality [34],and service development and commissioning is influenced via the data included in patient records [32]. This is where a clear, consistent definition for self-harm becomes valuable as it gives a shared reference point for staff and reduces the risk of interpretation which may contribute to these issues occurring. It is therefore important for patient safety and quality of care that self-harm is consistently and clearly defined across the health system.

The final area of findings for discussion is the inclusion of harm-reduction approaches. Standard 19 on harm-reduction was one of the most neglected standards throughout the policies (met by only three of 35 trusts). Harm reduction, also sometimes referred to as harm minimisation, is a contentious practice. The foundation of the harm reduction approach is a recognition that self-harm cessation may not be realistic with individuals for whom self-harm is an important coping mechanism [35]. For these individuals, efforts by staff within the inpatient environment to stop self-harm are sometimes instead met with an escalation in the severity of the patient’s self-harm. Where this coping mechanism is removed and not adequately replaced with an alternative, patients find increasingly hard to manage and riskier methods of self-harm to manage their distress and patients may therefore be provided with support which instead focuses on ensuring they self-harm in a way that poses the least amount of risk to their physical health as possible [36]. The contention surrounding harm-reduction approaches surrounds concerns for both the ethics and effectiveness of utilising such approaches [35,37].

A study by James et al. [38] on the views of inpatient staff on harm-reduction approaches demonstrates this. The authors highlighted differences in how staff who have used harm-reduction approaches and those who have not feel about their use. Staff members who had utilised harm-reduction approaches felt more positive towards their use, whereas those who had not utilised them expressed concern that the approach would make self-harm worse. The authors also reported that some staff fundamentally disagreed with the concept of harm-reduction, arguing that it directly contradicted their role and the role of the hospital in caring for those who self-harm. However, staff who had utilised harm-reduction approaches reported a decrease in the severity and incidence of self-harm on their wards. Similar benefits and concerns surrounding harm reduction approaches are expressed throughout other studies on the topic [35,39].

The findings of this study in relation to harm-reduction approaches could be a reflection of this contention. This contention, however, could be considered the most important reason to include clear guidance on such approaches in policy. Clear policy based on professional consensus gives staff a basis on which to act and has the ability to protect them during proceedings where their actions may be called into question [40,41]. Staff completing a survey surrounding supporting patients who self-harm by ligature [24] reported that they felt concern about being blamed by both the patient’s family (62.4%) and their workplace (46.8%) if a patient was to self-harm. The authors highlight the position this placed staff in whereby they felt pressure to take a more restrictive approach to care whilst also knowing the importance of supporting patient’s autonomy. Having a clear basis on which to act presented via policy has the potential to alleviate some of these concerns and allow staff to provide care which they feel is more effective and appropriate.

### Limitations and further areas for future research

There are several limitations which need to be taken into account in order to contextualise the findings of this study. The first of these is the comprehensiveness of the data. Ten (20%) of the trusts contacted either did not respond and therefore did not provide any data (n = 5) or withheld policies which they indicated met the inclusion criteria (n = 5). It is possible that the policies which were not able to be obtained may have impacted on the results had they been included in the analysis.

In addition to these ten trusts, a further seven trusts did not provide any policies meeting the inclusion criteria. These trusts may not have had any policies meeting the inclusion criteria, or the initial request made to trusts may have been interpreted in a way that meant policies eligible for inclusion were not disclosed. The request did not ask for the disclosure of a policies with any specific focus beyond the “management of self-harm or suicidal patients in inpatient settings” as initial internet searches highlighted variation in what trusts titled their policies and how they chose to divide this overarching topic into specific focuses, e.g., suicide prevention. Keeping this request broad may have meant some policies were not received that may have been eligible for inclusion. However, specifying a certain focus or specific title may have led to some policies which were titled or categorised differently being excluded too. It may also be the case that some policies were not disclosed by the trusts who did provide at least one eligible policy. It was determined that the potential insight into the accessibility of the information within the policies justified keeping the request broad.

It is also possible that trusts may have included information in policies which were not selected for inclusion that could have led to different results, particularly within those trusts which withheld certain policies due to patient safety concerns. However, trust policy portfolios are vast and to analyse every policy from every trust would not have been feasible. The categories and policies selected were agreed upon by the research team as a reflection of where the information was most likely to be held, and where staff were most likely to search for this information. Some trusts choosing to withhold certain policies due to patient safety concerns whilst some provided similarly named policies could be due to genuine differences in the contents of the policies. It could also be due to similar themes as those above, of differing interpretation by the team handling the request as to what ought to be provided in response to such a request. What one team view as a patient safety risk meaning information is not subject to disclosure under a Freedom of Information request, may not be seen as a risk by another. It is a limitation necessary to note that these five trusts may have had different results if these policies had been accessible.

A further limitation which needs to be taken into account is that some services only partially met standards due to the approach taken to analysis which meant that hundreds of points within the NICE guidelines were merged into just 20. Some trusts may have met some of the guidelines grouped into a single standard but not others within the same grouping and therefore were not marked as ‘meeting’ the standard as a whole. The findings of this study need to be contextualised in this way.

The approach to analysis also required the researcher extracting the data to interpret the contents of the policy and make a judgement on whether it reflected each standard. It may be possible that other individuals may have interpreted the policies differently which may have led to different results. The research team aimed to mitigate this risk by discussing those standards and policies which appeared to require a greater degree of interpretation.

It is also important to note that whilst the NICE guidelines were chosen due to their basis on expert consensus and an evidence-base, and remain widely accepted amongst practitioners as a representation of best-practice, this study does not draw conclusions on whether certain trusts have policies which reflect safe and effective care better than others. The guidelines do not make clear what evidence was considered or how the consensus is arrived at. The composition of the group of ‘experts’ is also unclear. The voice of those with lived experience in this is also unclear. They also do not specifically focus on inpatient mental health settings which may require a more nuanced approach to managing self-harm. They do, however, still cover this environment and remain the guidelines which are most widely recognised. They were therefore deemed the most appropriate guidelines to guide this study. However, future research should further investigate what ‘good’ practice looks like for managing self-harm in the inpatient environment before using this study to inform conclusions about how policy could amended to support the process of improving the management of self-harm.

Finally, this research does not allow conclusions to be drawn on whether the practices of frontline staff regarding the management of self-harm are reflecting trust policies and/or the NICE guidelines, and whether trust policy has an impact on staff practices. There is, however, emerging evidence that staff practices regarding the use of risk assessment tools continue to contradict NICE guidance. An investigation by the Health Services Safety Investigations Body (HSSIB) in 2024 [42] found evidence that English inpatient mental health services are continuing to use risk assessment tools and scales as part of their attempts to evaluate an individual’s risk of self-harm. The HSSIB report indicates that a contributory factor for staff choosing not to follow this part of the guideline is concerns that they may be blamed should incidents occur where a risk assessment and risk stratification has not taken place. Future research may wish to explore what role trust policy may play in influencing staff behaviours. As discussed, policy can be an important tool for giving staff a basis on which to act [40] and so policy could be one way of addressing this issue.

## Conclusion

This study has highlighted the variation in how English NHS mental health trusts approach the topic of the management of self-harm throughout their policy portfolio. Whether these policies improve the quality and safety of care is yet to be established. However, there is identifiable inconsistency in what guidance is presented to staff via policy and the format in which it is presented has the potential to cause confusion. Confusion and inconsistency have been shown to cause risks to quality and safety of care.

Future research may wish to consider what good practice looks like for the management of self-harm, beyond the NICE guidelines, by speaking directly with patients, carers, and staff. It may also be beneficial to investigate how staff utilise information sources to guide their practice as this could help inform the right format for sharing guidance clearly and consistently. Conducting a similar study within other international health systems may also help us to understand the relationship between national guidelines and organisational policy. Finally, research to help us further understand the impact that guidelines, policy, and other systemic factors have on staff practices could help us to determine where to target interventions to change staff practice and improve care.
